# Double-Layer Distributed and Integrated Fault Detection Strategy for Non-Gaussian Dynamic Industrial Systems

**DOI:** 10.3390/e26100815

**Published:** 2024-09-25

**Authors:** Shengli Dong, Xinghan Xu, Yuhang Chen, Yifang Zhang, Shengzheng Wang

**Affiliations:** 1 Merchant Marine College, Shanghai Maritime University, Shanghai 201306, China; dong.shengli@coscoshipping.com (S.D.); chen.yuhang@coscoshipping.com (Y.C.); szwang@shmtu.edu.cn (S.W.); 2 Shanghai Ship and Shipping Research Institute Co., Ltd., Shanghai 200135, China; 3 Faculty of Infrastructure Engineering, Dalian University of Technology, Dalian 116024, China; xuxh2023@dlut.edu.cn; 4 School of Control Science and Engineering, Dalian University of Technology, Dalian 116024, China

**Keywords:** fault detection, non-Gaussian dynamic systems, double-layer distributed, Laplacian score, integrated Bayesian inference

## Abstract

Currently, with the increasing scale of industrial systems, multisensor monitoring data exhibit large-scale dynamic Gaussian and non-Gaussian concurrent complex characteristics. However, the traditional principal component analysis method is based on Gaussian distribution and uncorrelated assumptions, which are greatly limited in practice. Therefore, developing a new fault detection method for large-scale Gaussian and non-Gaussian concurrent dynamic systems is one of the urgent challenges to be addressed. To this end, a double-layer distributed and integrated data-driven strategy based on Laplacian score weighting and integrated Bayesian inference is proposed. Specifically, in the first layer of the distributed strategy, we design a Jarque–Bera test module to divide all multisensor monitoring variables into Gaussian and non-Gaussian blocks, successfully solving the problem of different data distributions. In the second layer of the distributed strategy, we design a dynamic augmentation module to solve dynamic problems, a K-means clustering module to mine local similarity information of variables, and a Laplace scoring module to quantitatively evaluate the structural retention ability of variables. Therefore, this double-layer distributed strategy can simultaneously combine the different distribution characteristics, dynamism, local similarity, and importance of variables, comprehensively mining the local information of the multisensor data. In addition, we develop an integrated Bayesian inference strategy based on detection performance weighting, which can emphasize the differential contribution of local models. Finally, the fault detection results for the Tennessee Eastman production system and a diesel engine working system validate the superiority of the proposed method.

## 1. Introduction

With the continuous expansion of modern industrial production scale, increasingly complex industrial systems are more likely to cause major safety accidents. To ensure the long-term stable operation of industrial systems, it is necessary to accurately detect and diagnose faults in time [1,2,3,4,5]. With the rapid promotion of the Industrial Internet of Things, hundreds of millions of devices have generated massive numbers of multisensor monitoring data. Data-driven fault detection methods have attracted the attention of researchers [6,7,8,9,10]; especially, multivariate statistical methods are widely used, such as partial least squares [11] and principal component analysis (PCA) [12]. However, traditional static PCA assumes that the data follow a Gaussian distribution and the samples at different times are independent. Unfortunately, in actual industry, the above assumptions are not necessarily satisfied. Therefore, dynamic PCA (DPCA) [13,14] and slow feature analysis (SFA) [15] were proposed to solve the dynamic correlation problem. Independent component analysis (ICA) [16] and dynamic independent component analysis (DICA) [17,18,19] were proposed to deal with non-Gaussian monitoring data.

In fact, as industrial systems become increasingly complex, the number of monitoring variables collected gradually increases, exhibiting large-scale characteristics. Deep learning methods can effectively process industrial big data and have been widely applied in the field of industrial fault diagnosis [20,21,22]. However, in practical industrial applications, fault events rarely occur, making it difficult to collect sufficient fault datasets for deep learning model training. Moreover, the above methods input all variables into the model as a whole, which is essentially a centralized method that easily ignores the local characteristics of the multisensor monitoring data (such as distribution characteristics, dynamic correlations, local similarity). Unlike the centralized methods mentioned above, distributed methods first divide monitoring variables into conceptually meaningful subblocks based on their inherent characteristics and then establish local monitoring models, which can reduce system complexity, increase model flexibility, and reduce model misjudgment rate. Therefore, distributed methods have become a research hotspot for large-scale industrial system monitoring, such as distributed ICA [23,24,25], distributed SFA [26,27,28], distributed cointegration analysis [29,30], distributed canonical correlation analysis [31,32], and double-layer distributed process monitoring based on hierarchical multiblock decomposition (DL-MB) [33]. Although the above distributed methods have been proposed, distributed PCA [34,35,36] is still one of the most commonly used distributed fault detection methods.

Due to the complex distribution characteristics of multisensor monitoring variables in practical industrial systems, some variables follow a Gaussian distribution, while others do not, exhibiting both Gaussian and non-Gaussian concurrent characteristics. However, similar to PCA, distributed PCA also needs to satisfy Gaussian distribution and irrelevance assumptions, so it performs poorly in fault detection tasks for large-scale Gaussian and non-Gaussian concurrent dynamic systems. In addition, the complex relationships and importance of different variables, as well as the contribution of different local models to the overall model detection performance, all have a significant impact on the detection results of distributed methods. Therefore, addressing the key issues mentioned above can fully tap into the application potential of distributed PCA in large-scale Gaussian and non-Gaussian concurrent dynamic systems. Considering that block partitioning is the first and crucial step in establishing a distributed model, how can we design a block partitioning strategy that can isolate Gaussian and non-Gaussian variables, combine the dynamic and local similarity information of variables, and ultimately overcome the limitations of distributed PCA methods? How can we design a weighting strategy that can quantitatively evaluate the importance of different variables and local models, thereby significantly improving detection performance?

To our knowledge, there are currently no reports on fault detection in large-scale industrial systems from the perspectives of variable distribution characteristics, dynamism, local similarity, and the importance of variables and local models. Considering that the Jarque–Bera (J-B) test is an effective method for testing whether data follow a normal distribution, we first design a J-B test module to distinguish between Gaussian and non-Gaussian variables, and divide all Gaussian variables into a Gaussian block while dividing all non-Gaussian variables into a non-Gaussian block. Secondly, we design a dynamic augmentation module that adds previous observations to describe the dynamic correlation of variables. Next, we design the K-means module to cluster variables in both Gaussian and non-Gaussian blocks to mine local similarity information between variables. In addition, to evaluate the importance of different variables within the same cluster subblock, we calculate the Laplacian score (Ls) for each variable and use the score as the variable weight. The Ls algorithm calculates the score of the sample’s features, which can evaluate the local structure retention ability of different variables [37]. Additionally, we establish a PCA detection model in each Gaussian cluster subblock, and an ICA detection model in each non-Gaussian cluster subblock. Finally, the detection accuracy of each local detection model is used as a weight to obtain an integrated Bayesian inference strategy, ultimately obtaining global monitoring statistics for fault detection.

Based on the above discussion, a double-layer distributed and integrated fault detection method based on Laplacian score weighting and integrated Bayesian inference (LSW-IBI) is proposed for large-scale Gaussian and non-Gaussian concurrent dynamic systems.

The main contributions of this paper are as follows:

1. To solve the problem of low fault detection performance in industrial systems with complex-characteristic multisensor data, this paper designs a J-B test module to effectively separate Gaussian and non-Gaussian variables while designing a dynamic augmentation module to characterize the dynamic behavior of variables, overcoming the limitations of traditional PCA methods’ Gaussian distribution and time independence assumption, and significantly improving detection performance.
2. Compared to traditional single-layer distributed methods, we propose a new double-layer distributed framework. This framework develops an optimal block partitioning scheme based on the distribution characteristics and similarity of variables, which provides a new research approach for distributed fault monitoring.
3. This paper designs a Laplacian scoring weighting module and an integrated Bayesian inference module from the perspective of the differential importance of variable local structure retention ability and local model detection performance, providing a new dual weighting strategy that can quantitatively evaluate the contributions of different variables and local models.

The rest of this article is arranged as follows: Section 2 introduces PCA, ICA, and Laplacian score. Section 3 describes a double-layer distributed and integrated data-driven fault detection method. Section 4 discusses the application of the proposed methods in two cases, and the main conclusions are summarized in Section 5.

## 2. Related Work

### 2.1. Fault Detection Based on PCA

PCA is often used to reduce the dimensionality of data. Assuming a data matrix $X=\left[x_{1},x_{2},\ldots ,x_{m}\right]\in R^{n\times m}$ containing *n* samples and *m* variables, it can be expressed as follows:

(1) $$X=TP^{T}+E.$$

where X has been standardized, and $T\in R^{n\times k}$, $P\in R^{m\times k}$, and $E\in R^{n\times m}$ express the score matrix, load matrix, and residual matrix. The number of principal components *k* is obtained by calculating the cumulative variance percentage (CPV). The Hotelling’s T-squared ($T^{2}$) statistic and squared prediction error (SPE) statistic are expressed as follows:

(2) $$\begin{array}{c} T^{2}=t\Lambda_{k}^{-1}t^{T}\\ SPE=e\cdot e^{T}. \end{array}$$

where $\Lambda_{k}$ is composed of the first *k* largest eigenvalues of covariance matrix C. By calculating (3), the control limit can be obtained [25]:

(3) $$\begin{array}{c} T^{2}\leq \frac{k\left(n-1\right)}{n-k}F_{k,n-k,\alpha }\\ SPE\leq \theta_{1}{\left[\frac{C_{\alpha }\sqrt{2\theta_{2}h_{0}^{2}}}{\theta_{1}}+1+\frac{\theta_{2}h_{0}\left(h_{0}-1\right)}{\theta_{1}^{2}}\right]}^{\frac{1}{h_{0}}}. \end{array}$$

where $\theta_{i}=\sum_{j=k+1}^{m}\lambda_{j}^{i}\left(i=1,2,3\right)$, $h_{0}=1-\frac{2\theta_{1}\theta_{3}}{3\theta_{2}^{2}}$, $F_{k,n-k,\alpha }$ indicates the *F* distribution where the first degree of freedom and the second degree of freedom are *k* and n−k, respectively, and α denotes the significance level. $C_{\alpha }$ denotes the normal deviate corresponding to the upper 1−α percentile.

### 2.2. Fault Detection Based on ICA

Independent component analysis (ICA), which reveals higher-order statistical information from sensor data, is widely employed for dealing with non-Gaussian monitoring variables. Given a dataset $X={\left[x_{1},x_{2},\ldots ,x_{m}\right]}^{T}\in R^{m\times n}$ of *m* variables and *n* samples, X is decomposed into *d* unknown independent components (ICs):

(4) X=AS+E.

where $A\in R^{m\times d}$, $S={\left[s_{1},s_{2},\ldots ,s_{d}\right]}^{T}\in R^{d\times n}$, and E denote the mixed matrix, independent component matrix, and the residual matrix. In order to estimate A and S, $\hat{S}$ is decomposed by the FastICA method [17]:

(5) $$\hat{S}=WX.$$

First, for a given m-dimensional vector x, perform eigenvalue decomposition on its covariance matrix:

(6) $$C=E\left(xx^{T}\right)=U\Lambda U^{T}.$$

where $E\left(\cdot \right)$ denotes expectation, and U denotes the feature vector. The whitening operation is defined as follows:

(7) z=Qx.

where Q=Λ−1−122UT. Formula (7) is denoted as follows:

(8) z=QAs=Bs.

B is an orthogonal matrix that can be proved:

(9) $$E\left(zz^{T}\right)=BE\left(ss^{T}\right)B^{T}=BB^{T}=I.$$

From Formula (8), we can obtain the following:

(10) $$\hat{s}=B^{T}z=B^{T}Qx.$$

Then, the decomposition matrix is expressed as follows:

(11) $$W=B^{T}Q.$$

The $I^{2}$ statistic [17] and SPE statistic are constructed as follows:

(12) $$\begin{array}{c} I^{2}={\hat{s}}^{T}\hat{s}\\ SPE=e^{T}e={\left(x-\hat{x}\right)}^{T}\left(x-\hat{x}\right). \end{array}$$

where $\hat{s}=Wx$, $\hat{x}=Q^{-1}BWx$. The control limits of the above two statistics are calculated by using Kernel Density Estimation (KDE).

### 2.3. Laplacian Score

Laplacian score regards samples as nodes in a graph, and describes the similarity between different nodes (samples) through a similarity matrix (also called an adjacency matrix). On this basis, it evaluates the pros and cons of the features [37]. The Laplacian score is obtained by the following steps:

(1) Obtain the similarity matrix. If the *i*-th node $x_{i}$ and the *j*-th node $x_{j}$ are connected, the similarity between them can be expressed as follows:
  (13) $$S_{ij}=\exp\left(\frac{-{∥x_{i}-x_{j}∥}^{2}}{t}\right).$$
  where *t* is a suitable constant. If there is no connection between these two nodes, then $S_{ij}$ = 0. $S_{ij}$ represents the elements of the *i*-th row and *j*-th column in the similarity matrix S.
(2) Calculate the Laplacian matrix. Let $D=\mathrm{diag}\left(S1\right)$, where 1 represents a column vector with all 1 element, and the Laplacian matrix L=D−S.
(3) Calculate the score of the feature. Suppose there are *n* samples, and each sample has *m* features. $f_{ri}$ and $f_{rj}$ are the values of the *r*-th feature corresponding to the *i*-th sample and the *j*-th sample, respectively. $f_{r}={\left[f_{r1},f_{r2},\ldots ,f_{rm}\right]}^{T}$ is the *r*-th feature. A good feature should reflect the structure of the graph to the greatest extent. That is, if two points in the original data are similar, then the two points should also be similar under this feature. Select features by minimizing the following objective function:
  (14) $$L_{r}=\frac{\sum_{ij}{\left(f_{ri}-f_{rj}\right)}^{2}S_{ij}}{Var\left(f_{r}\right)}.$$
  For the above formula after algebraic calculation,
  (15) $$\begin{array}{c} \sum_{ij}{\left(f_{ri}-f_{rj}\right)}^{2}S_{ij}=\sum_{ij}{\left(f_{ri}^{2}{+f}_{rj}^{2}-2f_{ri}f_{rj}\right)}^{2}S_{ij}\\ =2\sum_{ij}f_{ri}^{2}S_{ij}-2\sum_{ij}f_{ri}S_{ij}f_{rj}\\ =2f_{r}^{T}Df_{r}-2f_{r}^{T}Sf_{r}=2f_{r}^{T}Lf_{r}\\ Var\left(f_{r}\right)=\sum_{i=1}^{n}{\left(f_{ri}-\mu \right)}^{2}D_{ii}. \end{array}$$
  where $\mu =\frac{f_{r}^{T}D1}{1^{T}D1}$ represents the sample mean of $f_{r}$. Through the normalization operation, the normalized vector ${\tilde{f}}_{r}$ can be obtained:
  (16) $${\tilde{f}}_{r}=f_{r}-\frac{f_{r}^{T}D1}{1^{T}D1}1.$$
  Then, $Var\left(f_{r}\right)$ can be estimated as follows:
  (17) $$Var\left(f_{r}\right)=\sum_{i}{\tilde{f}}_{ri}^{2}D_{ii}={\tilde{f}}_{r}^{T}D{\tilde{f}}_{r}.$$
  The Laplacian score of the *r*-th feature $f_{r}$ is represented by $L_{r}$. Let $S_{r}=\frac{{\tilde{f}}_{r}^{T}S{\tilde{f}}_{r}}{{\tilde{f}}_{r}^{T}D{\tilde{f}}_{r}}$, then
  (18) $$L_{r}=\frac{{\tilde{f}}_{r}^{T}L{\tilde{f}}_{r}}{{\tilde{f}}_{r}^{T}D{\tilde{f}}_{r}}=1-\frac{{\tilde{f}}_{r}^{T}S{\tilde{f}}_{r}}{{\tilde{f}}_{r}^{T}D{\tilde{f}}_{r}}=1-S_{r}.$$
  Generally, the smaller the Laplacian score, the better the feature. This article is achieved by maximizing $S_{r}$, that is, $S_{r}$ is used as the Laplacian score. If the score $S_{r}$ of $f_{r}$ is larger, then $f_{r}$’s ability to retain the local structure is stronger, and the greater the weight coefficient and contribution of $f_{r}$.

## 3. A Double-Layer Distributed and Integrated Data-Driven Fault Detection Method

### 3.1. First Layer Distributed Design: J-B Test Module

First, we divide all Gaussian variables into one subblock (group or subspace) and all non-Gaussian variables into another subblock by using the J-B test. In statistics, the J-B test is a goodness-of-fit test that tests whether the sensor data have skewness and kurtosis that conform to a normal distribution [30]. For a variable $x_{j}={\left[a_{1},a_{2},\ldots ,a_{n}\right]}^{T}$, calculate the skewness coefficient $\sqrt{s}$ and kurtosis coefficient *k*:

(19) $$\begin{array}{c} \sqrt{s}=\frac{\frac{1}{n}\sum_{i=1}^{n}{\left(a_{i}-\bar{a}\right)}^{3}}{{\left[\frac{1}{n}\sum_{i=1}^{n}{\left(a_{i}-\bar{a}\right)}^{2}\right]}^{\frac{3}{2}}},k=\frac{\frac{1}{n}\sum_{i=1}^{n}{\left(a_{i}-\bar{a}\right)}^{4}}{{\left[\frac{1}{n}\sum_{i=1}^{n}{\left(a_{i}-\bar{a}\right)}^{2}\right]}^{2}}. \end{array}$$

When $x_{j}$ obeys Gaussian distribution, $\sqrt{s}$ is close to 0, and *k* is close to 3. The Jarque–Bera (J-B) statistic can be expressed as follows:

(20) $$\mathrm{JB}=\frac{n}{6}\left[{\left(\sqrt{k}\right)}^{2}+\frac{{\left(s-3\right)}^{2}}{4}\right].$$

All data $X=\left[x_{1},x_{2},\ldots ,x_{m}\right]\in R^{n\times m}$ with *n* samples and *m* variables are grouped into a Gaussian block $X^{G}$ and a non-Gaussian block $X^{NG}$, where $X=\left[X^{G},X^{NG}\right]$.

### 3.2. Second Layer Distributed Design

(1) Dynamic augmentation module

In real industrial system monitoring, variables collected by multisensor methods tend to have autocorrelated dynamic properties. Therefore, we design a dynamic augmentation module based on the idea of time delay displacement to solve the dynamic problem. Specifically, first, we expand the Gaussian block $X^{G}$ to $X_{a}^{G}$ by adding observations of the first *l* moments:

(21) $$X_{a}^{G}=\left[\begin{array}{cccc} x_{1+l}^{T} & x_{l}^{T} & \cdots & x_{1}^{T}\\ x_{2+l}^{T} & x_{1+l}^{T} & \cdots & x_{2}^{T}\\ \vdots & \vdots & \ddots & \vdots \\ x_{n}^{T} & x_{n-1}^{T} & \cdots & x_{n-l}^{T} \end{array}\right]$$

For details of the selection of time lag parameters *l*, see [13]. In most processes, *l* can be set to 1 or 2 [17]. Similarly, we dynamically augment non-Gaussian block $X^{NG}$ to $X_{a}^{NG}$.

(2) K-means clustering module

Then, the K-means method [38] is used to cluster the variables with strong similarity in the above augmented Gaussian block $X_{a}^{G}$ and non-Gaussian block $X_{a}^{NG}$, respectively. The optimization objective of K-means is to minimize the sum of squared errors between each sample and the cluster center:

(22) $$\min\sum_{j=1}^{c}\sum_{x_{i}\in \Gamma_{j}}{\left|x_{i}-\tau_{j}\right|}^{2}.$$

where *c* represents the number of clusters, $\tau_{j}$ represents the center of the *j*-th cluster, and $\Gamma_{j}$ represents the set of vectors in the *j*-th cluster. If *c* is not an optimal number, the results of the clustering may not be optimal. The number is determined by the essential characteristics of the dataset, which is derived by the objective of maximizing the cluster distance and minimizing the within-cluster distance. If the final center of the *j*-th cluster is expressed as $\tau_{j}^{*}$, the optimization index *L* [39] is expressed as follows:

(23) $$L=\frac{\frac{1}{m}\sum_{j=1}^{c}\sum_{x_{i}\in \Gamma_{j}}{\left|x_{i}-\tau_{j}^{*}\right|}^{2}}{\frac{2}{c(c-1)}\sum_{i=1}^{c}\sum_{j=i+1}^{c}{\left|\tau_{i}^{*}-\tau_{j}^{*}\right|}^{2}}$$

where *m* is the number of variables in $\Gamma_{j}$. When the index *L* reaches the minimum, the optimal number of clusters is determined. According to the clustering results of the training dataset, the Gaussian block is split into *h* clustering subblocks, $X_{a}^{G}=\left[X_{1}^{G},X_{2}^{G},\ldots ,X_{h}^{G}\right]$, and the non-Gaussian block is split into *b* clustering subblocks, $X_{a}^{NG}=\left[X_{1}^{NG},X_{2}^{NG},\ldots ,X_{b}^{NG}\right]$.

(3) Laplacian score weighting module

$f_{r}^{i}$$\left(r=1,\ldots ,m_{i}\right)$ represents the *r*-th measured variable in the *i*-th Gaussian clustering subblock $X_{i}^{G}$, $m_{i}$ represents the total number of variables in $X_{i}^{G}$.

Calculate the Laplacian score $S_{r}^{i}$ of $f_{r}^{i}$ using (24):

(24) $$S_{r}^{i}=\frac{{\left({\tilde{f}}_{r}^{i}\right)}^{T}L{\tilde{f}}_{r}^{i}}{{\left({\tilde{f}}_{r}^{i}\right)}^{T}D{\tilde{f}}_{r}^{i}},\;{\tilde{f}}_{r}^{i}=f_{r}^{i}-\frac{{\left(f_{r}^{i}\right)}^{T}D1}{1^{T}D1}1$$

Then, normalize the score by ${\tilde{S}}_{r}^{i}=S_{r}^{i}/\sum_{r=1}^{m_{i}}S_{r}^{i}$. Therefore, we obtain the weighted measured variable ${\tilde{f}}_{r}^{i}={\tilde{S}}_{r}^{i}f_{r}^{i}$. For simplicity, we still mark the weighted Gaussian clustering subblock as $X_{i}^{G}$. In the same way, the weighted non-Gaussian clustering subblock $X_{i}^{NG}$ can be obtained.

### 3.3. Integrated Bayesian Inference Module

Establish a PCA model in each weighted Gaussian cluster subblock. For the *i*-th Gaussian cluster subblock $X_{i}^{G}$,

(25) $$X_{i}^{G}=T_{i}P_{i}^{T}+E_{i}.$$

Establish an ICA model in each weighted non-Gaussian cluster subblock. For the *j*-th non-Gaussian cluster subblock $X_{j}^{NG}$,

(26) $$X_{j}^{NG}=A_{j}S_{j}+E_{j}.$$

The $T_{i}^{2}$ and ${SPE}_{i}$ statistics are obtained using (2). The $I_{j}^{2}$ and ${SPE}_{j}$ statistics are obtained using (12). For a given sample $x=\left[x_{1}^{G},x_{2}^{G},\ldots ,x_{h}^{G},x_{1}^{NG},x_{2}^{NG},\ldots ,x_{b}^{NG}\right]$, under the $T_{i}^{2}$ statistic, the fault probability of $x_{i}^{G}$ is expressed as follows:

(27) $$P_{T^{2}}\left(\left.F\right|x_{i}^{G}\right)=\frac{P_{T^{2}}\left(\left.x_{i}^{G}\right|F\right)P_{T^{2}}\left(F\right)}{P_{T^{2}}\left(x_{i}^{G}\right)}$$

Let $p_{T^{2}}\left(x_{i}^{G}\right)=p_{T^{2}}\left(\left.x_{i}^{G}\right|N\right)p_{T^{2}}\left(N\right)+p_{T^{2}}\left(\left.x_{i}^{G}\right|F\right)p_{T^{2}}\left(F\right)$, where *N* and *F* denote normal and fault states, $P_{T^{2}}\left(N\right)$ is equal to the confidence level α, and $P_{T^{2}}\left(F\right)$ is equal to 1−α. The class conditional probability of $x_{i}^{G}$ under the $T^{2}$ statistic can be expressed as follows:

(28) PT2xiGN=exp−Ti2−Ti2Tlimi2Tlimi2.

(29) PT2xiGF=exp−Tlimi2−Tlimi2Ti2Ti2.

where $T_{i}^{2}$ and $T_{limi}^{2}$ represent statistics and thresholds of $x_{i}^{G}$, respectively. The same can obtain $P_{I^{2}}\left(\left.F\right|x_{j}^{NG}\right)$. We define the detection accuracy rate as the proportion of the number of samples with consistent predicted and real categories to the total number of samples. Then, the normalized detection accuracy rates of $x_{i}^{G}$ and $x_{j}^{NG}$ are defined as follows:

(30) $$w_{i}^{T^{2}}=\frac{{\hat{w}}_{i}^{T^{2}}}{\sum_{i=1}^{h}{\hat{w}}_{i}^{T^{2}}+\sum_{j=1}^{b}{\hat{w}}_{j}^{I^{2}}},\;w_{j}^{I^{2}}=\frac{{\hat{w}}_{j}^{I^{2}}}{\sum_{i=1}^{h}{\hat{w}}_{i}^{T^{2}}+\sum_{j=1}^{b}{\hat{w}}_{j}^{I^{2}}}$$

where ${\hat{w}}_{i}^{T^{2}}{=Acc}_{i}^{T^{2}}$ and ${\hat{w}}_{j}^{I^{2}}{=Acc}_{j}^{I^{2}}$ represent the sample detection accuracy rate of $x_{i}^{G}$ and $x_{j}^{NG}$, respectively. Finally, we assign the normalized detection accuracy of each clustering subblock as the weight of its fault probability index, and fuse the fault detection results of all subblocks into a global monitoring statistical index $BIC\text{-}C^{2}$ based on the Bayesian Information Criterion (BIC) as shown in (31). In the same way, the BIC-SPE statistic can be obtained. In the actual detection process, as long as one of the $BIC\text{-}C^{2}$ and BIC-SPE statistical indicators exceeds the threshold 1−α, it means that a fault has occurred in the current process and corresponding measures should be taken.


(31) $$BIC\text{-}C^{2}=\frac{\sum_{i=1}^{h}w_{i}^{T^{2}}P_{T^{2}}\left(\left.x_{i}^{G}\right|F\right)P_{T^{2}}\left(\left.F\right|x_{i}^{G}\right)+\sum_{j=1}^{b}w_{j}^{I^{2}}P_{I^{2}}\left(\left.x_{j}^{NG}\right|F\right)P_{I^{2}}\left(\left.F\right|x_{j}^{NG}\right)}{\sum_{i=1}^{h}w_{i}^{T^{2}}P_{T^{2}}\left(\left.x_{i}^{G}\right|F\right)+\sum_{j=1}^{b}w_{j}^{I^{2}}P_{I^{2}}\left(\left.x_{j}^{NG}\right|F\right)}$$

**Figure 1:**
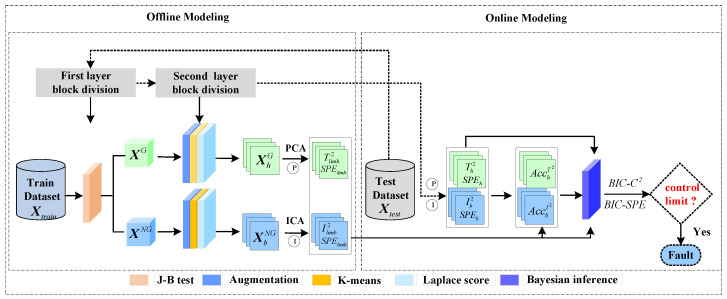
The flowchart of the double-layer distributed and integrated fault detection strategy based on LSW-IBI.

The proposed LSW-IBI algorithm is shown in Algorithm 1. A flowchart of the double-layer distributed and integrated fault detection strategy based on LSW-IBI is shown in Figure 1.

**Remark 1.**  *The developed double-layer distributed and integrated fault detection strategy includes five modules: the J-B test module, dynamic augmentation module, K-means clustering module, Laplacian score module, and integrated Bayesian inference module. Compared with existing methods, this strategy fully considers the different distribution characteristics, dynamic correlation, local similarity, and local structure retention ability (importance) of multisensor data through the first four modules designed, expanding the depth of traditional single-layer distributed methods and comprehensively mining the local information of data. The integrated Bayesian inference module highlights the impact of local model detection performance on the global monitoring model, effectively reducing the uncertainty brought by the local model fusion process.*

**Algorithm 1:** LSW-IBI
**Offline modeling**:
**Step (1)** Standardize training dataset $X_{train}$.
**Step (2)** By J-B test module divide monitoring variables into Gaussian block $X^{G}$ and non-Gaussian block $X^{NG}$.
**Step (3)** Apply dynamic augmentation module, K-means clustering module, and Laplace score weighting module to $X^{G}$ and $X^{NG}$ to obtain subblocks ${\left\{X_{i}^{G}\right\}}_{i=1}^{h}$ and ${\left\{X_{j}^{NG}\right\}}_{j=1}^{b}$.
**Step (4)** Establish PCA model for Gaussian clustering subblock ${\left\{X_{i}^{G}\right\}}_{i=1}^{h}$, and establish ICA model for non-Gaussian clustering subblock ${\left\{X_{j}^{NG}\right\}}_{j=1}^{b}$ to obtain control limits ${\left\{T_{limi}^{2},SPE_{limi}\right\}}_{i=1}^{h}$ and ${\left\{I_{limj}^{2},SPE_{limj}\right\}}_{j=1}^{b}$.
**Online modeling**:
**Step (1)** Standardize test set $X_{test}$, and use the partition rules in step 2 and step 3 of the offline phase to obtain test set Gaussian and non-Gaussian clustering subblocks.
**Step (2)** Establish monitoring model to obtain statistics ${\left\{T_{i}^{2},SPE_{i}\right\}}_{i=1}^{h}$ and ${\left\{I_{j}^{2},SPE_{j}\right\}}_{j=1}^{b}$. Calculate accuracy rate ${\left\{Acc_{i}^{T^{2}}\right\}}_{i=1}^{h}$ and ${\left\{Acc_{j}^{I^{2}}\right\}}_{j=1}^{b}$ by the control limit in step 4 of the offline phase, then normalized them by (30).
Step (3) Obtain the statistics $BIC\text{-}C^{2}$ and BIC-SPE, and compare with threshold 1−α.


## 4. Case Study

### 4.1. TE Production System

**Figure 2:**
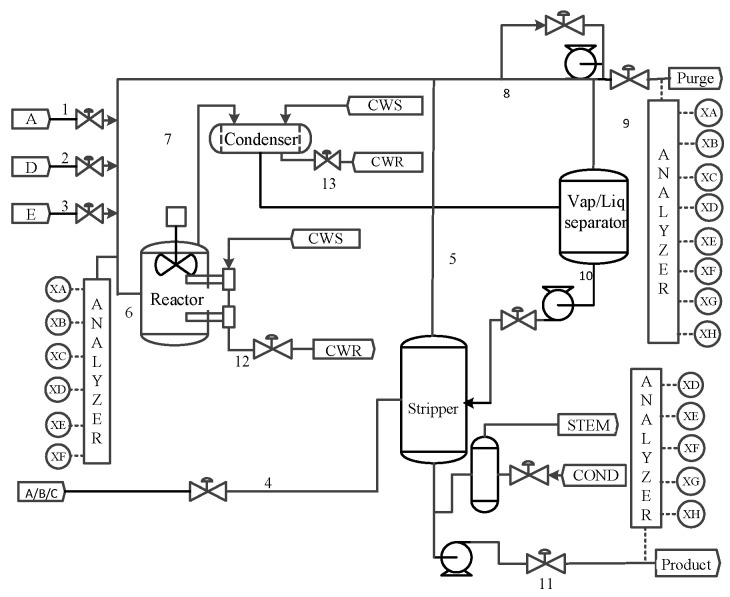
The flowchart of TE benchmark process [30].

The TE process is a benchmark simulation created by Downs et al. [40] based on a real chemical production system. The TE system mainly includes five main components, namely, the reactor, separator, product condenser, compressor, and product stripper [25,26]. The control system is shown in Figure 2. See [30] for a detailed description of the TE sensor monitoring variables. We consider the first 52 variables and train using a dataset containing 960 normal samples. We use all 960 samples from each fault simulation dataset for testing. Since the fault is introduced in the 161st observation, the first 160 samples are normal samples and samples 161–960 are fault samples. All fault data are standardized using the mean and variance of samples under normal operating conditions before use. The confidence level of all methods is set to 97%. The significance level of the J-B test is set to 0.05.

**Figure 3:**
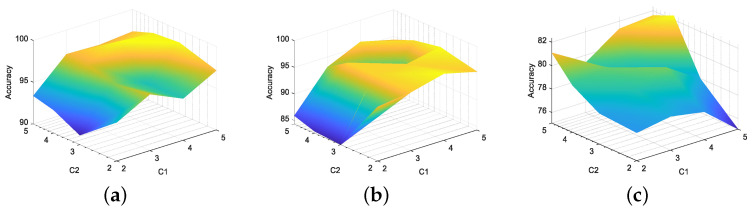
*l* = 1. Accuracy under different number of clusters: (**a**) fault 11, (**b**) fault 19, (**c**) fault 20.

**Figure 4:**
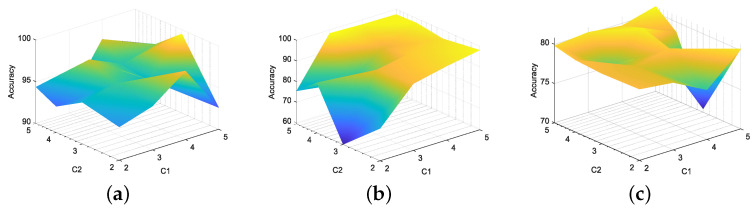
*l* = 2. Accuracy under different number of clusters: (**a**) fault 11, (**b**) fault 19, (**c**) fault 20.

**Figure 5:**
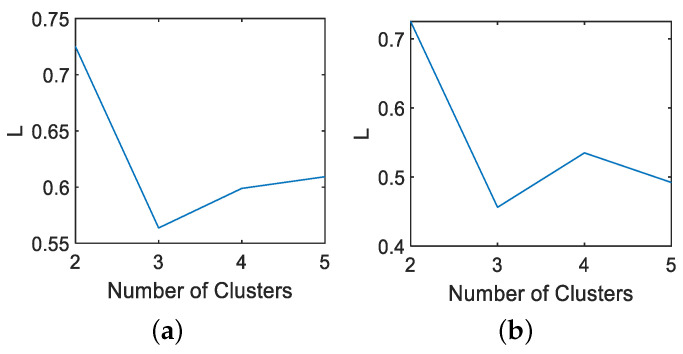
Optimal number of clusters: (**a**) Gaussian block, (**b**) non-Gaussian block.

**Figure 6:**
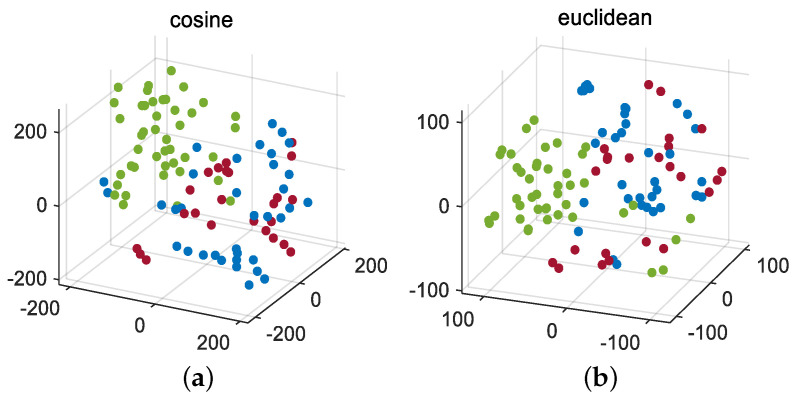
T-SNE for Gaussian block: (**a**) cosine, (**b**) Euclidean.

**Figure 7:**
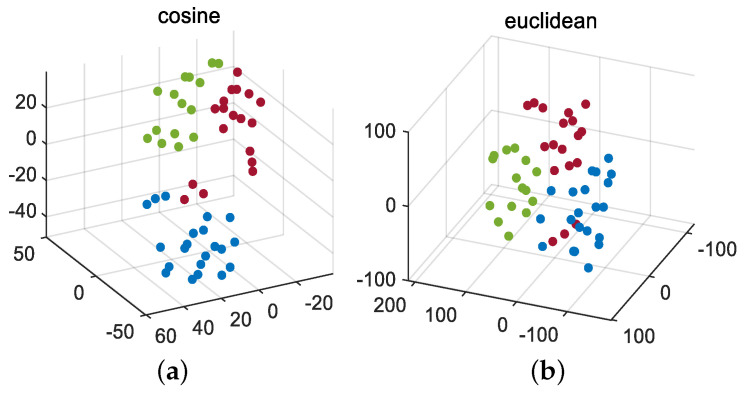
T-SNE for non-Gaussian block: (**a**) cosine, (**b**) Euclidean.

Firstly, the J-B test is conducted on 52 monitoring variables, and the results are shown in Table 1. Divide all Gaussian variables into a Gaussian block and all non-Gaussian variables into a non-Gaussian block using dynamic augmentation for variables in Gaussian and non-Gaussian blocks. In reference [13], it was proven that, when the lag parameter *l* value is equal to 1 or 2, it is sufficient to describe the dynamic characteristics of most processes. Therefore, consistent with [13,14,17], we choose the same lag order of *l* = 2. Next, the K-means clustering method is used to cluster the variables in the augmented Gaussian and non-Gaussian blocks, respectively, to obtain the corresponding Gaussian and non-Gaussian clustering subblocks. To further verify the robustness of the proposed method to the lag parameter *l* and the number of clusters, we fix *l* = 1 and *l* = 2 and simulate the detection accuracy of faults 11, 19, and 20 under different Gaussian cluster numbers C1 and non-Gaussian cluster number C2. The visualization results are shown in Figure 3 and Figure 4. Considering the complexity of offline modeling, both C1 and C2 values are selected within the range of [2, 5]. As can be seen from Figure 3 and Figure 4, in terms of the BIC-SPE statistic, whether *l* = 1 or *l* = 2, the proposed method can achieve high accuracy for all three types of faults under different cluster numbers. Therefore, the proposed method is robust to different lag parameters and cluster number parameters. Then, to select the optimal number of clusters for Gaussian and non-Gaussian blocks, using Equation (23), we obtain the relationship between the different number of clusters and the optimization index *L* value, as shown in Figure 5. From Figure 5, it can be seen that, for both Gaussian and non-Gaussian blocks, the *L* value reaches its minimum when the number of clusters is 3. Therefore, the optimal number of clusters for Gaussian and non-Gaussian blocks is set to 3. To further observe whether the data can be separated in low dimensions, Gaussian and non-Gaussian blocks are projected into three-dimensional space using the T-Distributed Stochastic Neighbor Embedding (T-SNE) method [41], and the visualization results are shown in Figure 6 and Figure 7. For Gaussian blocks, the 3D embedding losses calculated using cosine distance and Euclidean distance are 0.190 and 0.179, respectively. For non-Gaussian blocks, the 3D embedding losses calculated using cosine distance and Euclidean distance are 0.092 and 0.078, respectively. Obviously, embedding the data into low dimensional space using T-SNE will not lose too much original information. In Figure 6 and Figure 7, the clustering phenomenon in low-dimensional space is very obvious for both Gaussian and non-Gaussian blocks, indicating that it is reasonable to divide Gaussian and non-Gaussian blocks into three categories.

Table 2 shows the Fault Detection Rate (FDR) of PCA, DPCA [13], DWPCA [14], DICA [17], DPCA-DICA [25], DL-MB [33], and the proposed LSW-IBI method. It can be seen that all methods have good detection results for faults 1, 2, 4, 6, 7, 8, 12, 13, 14, and 18. Because faults 3, 9, and 15 are difficult to detect, their highest FDRs are only 0.308, 0.266, and 0.450, respectively. For $BIC\text{-}C^{2}$ and BIC-SPE statistics, the LSW-IBI method can achieve the highest detection rate for faults 1, 2, 4, 6, 7, 8, 10, 11, 12, 13, 14, 15, 17, 18, 19, and 20, indicating that it is widely applicable to different faults. For the BIC-SPE statistic, the average FDR of the proposed method is higher than that of other methods, reaching a maximum value of 0.776, indicating its outstanding detection performance. To intuitively observe the detection performance of different methods, we analyze faults 11 and 19 in detail.

**Figure 8:**
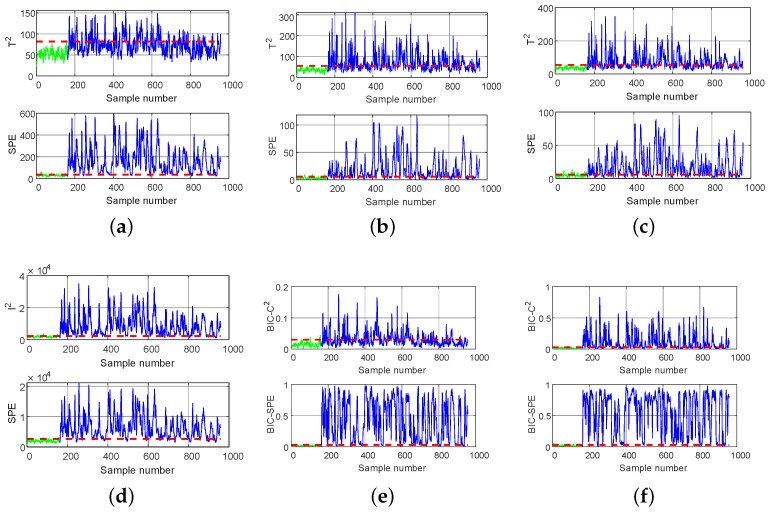
Monitoring charts of fault 11: (**a**) PCA, (**b**) DPCA, (**c**) DWPCA, (**d**) DICA, (**e**) DPCA-DICA, (**f**) LSW-IBI.

Fault 11 is the random variation of the reactor cooling inlet temperature. Due to its complexity, process data are more likely to show different distribution characteristics and dynamic characteristics. The detection results for fault 11 obtained by the six methods are shown in Figure 8. It can be seen that, under the $T^{2}$ statistic, PCA and DPCA find it difficult to detect faults, with a low FDR of 0.206 and 0.371, respectively. The detection rate of DWPCA is 0.662, slightly higher than that of PCA and DPCA, which may be due to DWPCA considering both the dynamic correlation of variables and the weight of the principal components. It is found that DICA (0.916) is superior to PCA, DPCA, and DWPCA because the DICA takes advantage of the high-order statistics to extract independent components while PCA-based methods use only the mean value and variance to extract unrelated components. However, DPCA-DICA (0.917) considers the different distribution characteristics of monitoring data, so its detection performance is superior to that of DICA (0.892) under the BIC-SPE statistic. Due to the use of a double-layer detection method based on hierarchical multiblock decomposition in DL-MB, its DR statistic [33] (0.933) achieves a suboptimal value. The proposed LSW-IBI has the best detection performance, and, under the BIC-SPE statistic, the FDR value reaches a maximum value of 0.949.

**Figure 9:**
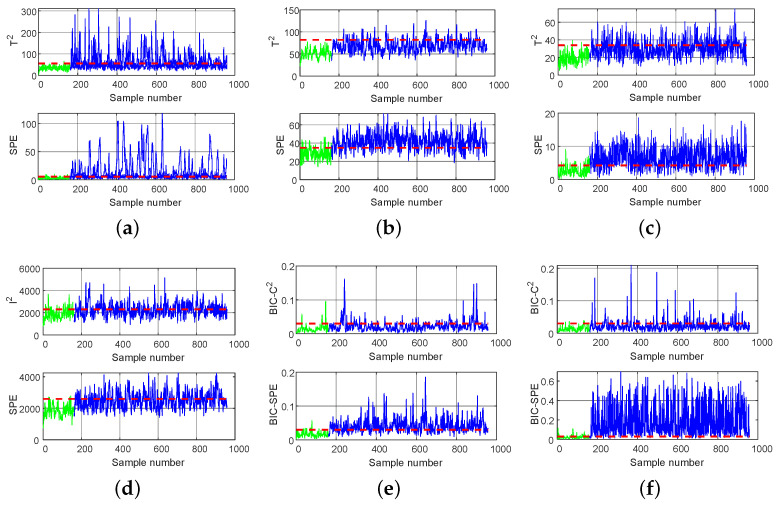
Monitoring charts of fault 19: (**a**) PCA, (**b**) DPCA, (**c**) DWPCA, (**d**) DICA, (**e**) DPCA-DICA, (**f**) LSW-IBI.

Fault 19 is an unknown fault. The detection results of fault 19 are shown in Figure 9. It can be seen that all methods cannot detect faults well under the $I^{2}$, $T^{2}$, and $BIC\text{-}C^{2}$ statistics. In terms of the SPE, DR, and BIC-SPE statistics, the FDR of the proposed method is significantly higher than that of the comparison methods, and the numerical order from high to low is LSW-IBI (0.930), DL-MB (0.847), DWPCA (0.823), DPCA (0.711), DPCA-DICA (0.678), DICA (0.419), and PCA (0.163). Because each method considered has two indices, which may give different results, it is difficult to say which is more effective due to the different characteristics and advantages of DWPCA compared to DICA and DPCA-DICA. Therefore, this is not contradictory to the analysis of fault 11. However, compared to other methods, the quantitative results can fully demonstrate the feasibility and effectiveness of the LSW-IBI method. The main reason is that LSW-IBI adopts five designed modules that can simultaneously combine the distribution characteristics, dynamism, local similarity, and importance of variables, as well as the detection performance information of local models.

### 4.2. A Real Diesel Engine Working System

**Figure 10:**
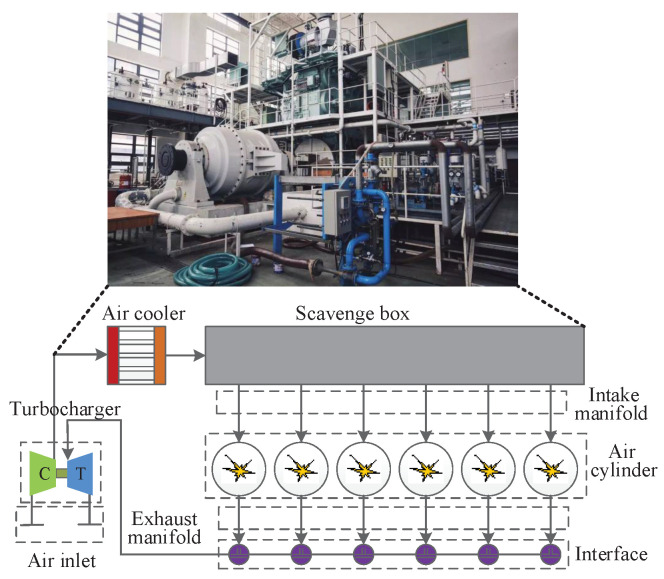
The entity and structure diagram of the diesel engine.

Diesel engines are widely used in marine power equipment and are prone to faults during operation. In this section, the faults occurring in a working system of a 6S35ME-B9 diesel engine are analyzed to further verify the fault detection performance of the proposed method. An entity diagram and structure diagram of the diesel engine are shown on the upper and lower sides of Figure 10. Six-cylinder double-stroke diesel engines mainly include turbochargers, air coolers, scavenging boxes, and cylinders. The collected data include 15 different sensor monitoring variables with a sampling interval of 10 s. See [36,42] for details of all variables. We use 969 normal samples for training, and select 345 fault samples for testing. Different from in the TE production system, the test data introduce faults from the first sample.

**Figure 11:**
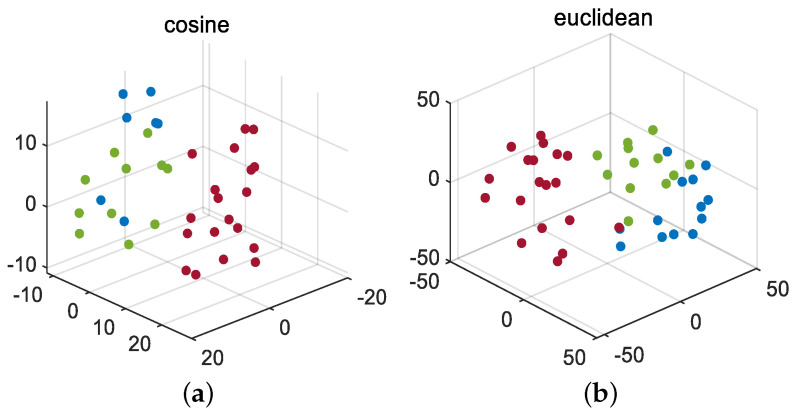
T-SNE for diesel engine: (**a**) cosine, (**b**) Euclidean.

First, we carry out the J-B test on 15 monitoring variables under the normal working conditions of the diesel engine. The test results show that all monitoring variables do not obey Gaussian distribution. This may depend on the actual operating conditions of the diesel engine, resulting in strong non-Gaussian characteristics of monitoring variables. Therefore, we directly dynamically expand the non-Gaussian variables and then perform K-means clustering. We use the same method as in the TE experiment and obtain the optimal number of clusters for the monitoring variables, which is 3. Similarly, the non-Gaussian block is projected into 3D space by the T-SNE method. The clustering visualization results are shown in Figure 11. The 3D embedding loss values based on the cosine distance and Euclidean distance are 0.119 and 0.084, respectively. Obviously, using T-SNE to embed these data into low-dimensional space does not lose much information. It can be seen from Figure 11 that the phenomenon of low-dimensional clustering is obvious, so it is reasonable to set the number of clusters to be equal to 3. Next, we assign standardized Laplacian scores to weight variables. Finally, the ICA model is established for each cluster subblock, and the corresponding detection statistics are obtained by integrated Bayesian inference method.

In this section, we combine the False Alarm Rate (FAR) to comprehensively evaluate the detection performance of different methods. Specifically, we test 960 normal samples to obtain the FAR and 345 exhaust pipe blockage fault samples to obtain the FDR. It is worth noting that, unlike in the TE experiment, we also compare the detection performance of three non-Gaussian clustering subblocks (i.e., block1, block2, block3) obtained through K-means clustering. The FAR and FDR values of DPCA, DICA, and the proposed method are shown in Table 3.

From Table 3, it can be seen that, for normal samples, under the $T^{2}$ and $I^{2}$ statistics, the FAR values of each method do not differ significantly. For the SPE statistic, the DICA method achieves the minimum FAR value of 0.031. Although the LSW-IBI method’s value is slightly higher than that of other comparison methods, it is still within an acceptable range. For the exhaust pipe blockage faults, under the same statistics, the FDR value of the DICA method (0.706/0.824) is slightly higher than that of the DPCA method (0.758/0.860), verifying that the DICA method is more suitable for non-Gaussian monitoring data. The DR statistic value of DL-MB is 0.862, which is better than for DPCA and DICA, but lower than for the proposed LSW-IBI method (0.919). The FDR of the LSW-IBI method reaches its maximum value (0.817/0.919), significantly higher than that of DPCA and DICA, fully demonstrating that the proposed method can significantly improve the detection performance for this fault. Under the $I^{2}$ and SPE statistics, the FDR of block2 (0.805/0.869) and block3 (0.698/0.869) is higher than that of block1 (0.564/0.773). The three subblocks have different FDR values, indicating that there are certain differences in the detection performance of each local model. Therefore, using different local models may lead to inconsistent conclusions, indicating that the detection results have significant uncertainty. Due to the use of a detection performance weighting strategy in the LSW-IBI method, which highlights the contributions of different local models, the detection results of the integrated model are significantly better than those of each subblock.

**Figure 12:**
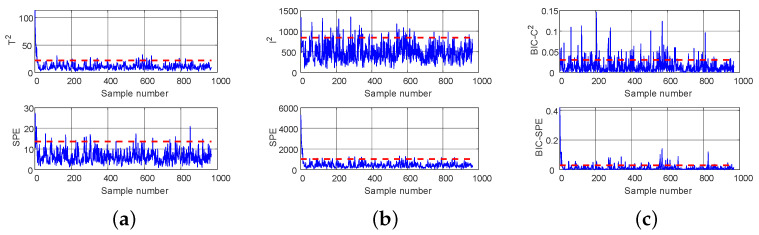
Detection of normal samples: (**a**) DPCA, (**b**) DICA, (**c**) LSW-IBI.

**Figure 13:**
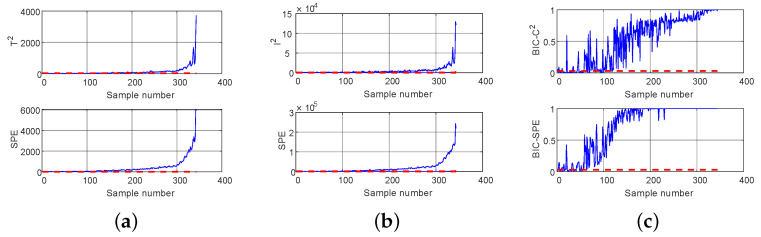
Detection of fault samples: (**a**) DPCA, (**b**) DICA, (**c**) LSW-IBI.

The detection results of DPCA, DICA, and LSW-IBI methods for normal samples and exhaust pipe blockage fault samples are shown in Figure 12 and Figure 13, respectively. It can be seen that the LSW-IBI method is superior to other comparison methods in detecting exhaust pipe blockage fault, and its false alarm probability for normal samples is still within the acceptable range, which is consistent with the results in Table 3. Therefore, the proposed method has practical application value for real marine diesel engine working systems.

## 5. Conclusions

This paper develops a double-layer distributed and integrated fault detection method for large-scale Gaussian and non-Gaussian concurrent dynamic systems. Specifically, by designing a J-B test module, dynamic augmentation module, K-means clustering module, Laplacian scoring weighting module, and integrated Bayesian inference module, important local information such as the variable distribution characteristic, dynamic characteristic, local similarity, importance, and difference contained in multisensor monitoring data is fully explored, ultimately achieving a significant improvement in fault detection performance. When all monitoring variables follow a Gaussian distribution, the proposed model degenerates into a distributed DWPCA model. When only implementing the J-B test module and dynamic augmentation module, it degenerates into a single-layer distributed DPCA-DICA model. The proposed method is a generalization of all comparison methods; in other words, all comparison methods are special cases of the proposed method. Therefore, the proposed method has wider applicability and can be applied to most scenarios in practical industrial systems. From an experimental perspective, it can be observed from the simulation results on the TE process and a real marine diesel engine working process that the proposed method achieves more satisfactory detection results compared to other methods.

## Figures and Tables

**Table 1:** J-B test results of monitoring variables.

	Gaussian Variables	Non-Gaussian Variables
Variable no.	1, 4, 5, 6, 8, 9, 11, 12, 14, 15, 17, 20, 21	2, 3, 7, 10, 13, 16
	22, 23, 24, 25, 26, 27, 29, 30, 35, 36	32, 33, 34, 39, 41, 46
	37, 38, 40, 42, 43, 44, 45, 48, 49, 51, 52	47, 50, 18, 19, 28, 31

**Table 2:** FDR achieved by different monitoring methods for TE process.

Fault No.	PCA		DPCA		DWPCA		DICA		DPCA-DICA		DL-MB	LSW-IBI	
	$T^{2}$	SPE	$T^{2}$	SPE	$T^{2}$	SPE	$I^{2}$	SPE	$BIC\text{-}C^{2}$	BIC-SPE	DR	$BIC\text{-}C^{2}$	BIC-SPE
1	0.993	0.999	0.995	**1.000**	0.995	**1.000**	**1.000**	0.998	0.999	0.998	0.999	**1.000**	**1.000**
2	0.986	0.985	0.989	0.986	0.989	0.987	0.991	0.988	0.989	0.990	0.992	**0.996**	0.994
3	0.040	0.049	0.029	0.163	0.201	0.175	**0.308**	0.105	0.168	0.118	0.184	0.216	0.123
4	0.504	**1.000**	0.135	**1.000**	0.512	**1.000**	**1.000**	**1.000**	0.196	**1.000**	**1.000**	0.929	**1.000**
5	0.284	0.315	0.276	0.530	0.262	**0.558**	0.513	0.336	0.350	0.456	0.412	0.425	0.434
6	0.990	**1.000**	0.994	**1.000**	0.994	**1.000**	0.998	0.995	0.998	**1.000**	0.996	0.998	**1.000**
7	**1.000**	**1.000**	**1.000**	**1.000**	**1.000**	**1.000**	**1.000**	**1.000**	**1.000**	**1.000**	**1.000**	**1.000**	**1.000**
8	0.974	0.973	0.975	0.979	0.975	0.981	0.984	0.981	0.981	0.984	0.985	**0.990**	0.989
9	0.049	0.035	0.020	0.175	0.150	0.174	**0.266**	0.119	0.135	0.130	0.171	0.186	0.132
10	0.356	0.418	0.397	0.561	0.504	0.608	0.635	0.485	0.555	0.285	0.624	**0.813**	0.712
11	0.206	0.675	0.371	0.935	0.662	0.939	0.916	0.892	0.391	0.917	0.933	0.691	**0.949**
12	0.988	0.950	0.994	0.976	0.994	0.982	**1.000**	0.995	0.999	0.991	0.998	**1.000**	0.995
13	0.949	0.950	0.951	0.957	0.951	0.960	0.962	0.954	0.960	0.954	0.958	0.961	**0.975**
14	0.998	**1.000**	**1.000**	**1.000**	**1.000**	**1.000**	**1.000**	**1.000**	0.986	**1.000**	**1.000**	**1.000**	**1.000**
15	0.039	0.065	0.038	0.160	0.238	0.173	0.291	0.119	0.160	0.119	0.223	0.227	**0.450**
16	0.183	0.401	0.193	0.553	0.174	**0.600**	0.508	0.318	0.410	0.209	0.519	0.525	0.486
17	0.809	0.955	0.820	0.976	0.901	0.976	0.937	0.914	0.836	0.975	0.964	0.947	**0.983**
18	0.896	0.908	0.897	0.917	0.916	0.921	0.941	0.897	0.901	0.906	0.926	0.951	**0.955**
19	0.183	0.163	0.259	0.711	0.396	0.823	0.435	0.419	0.183	0.678	0.847	0.184	**0.930**
20	0.354	0.548	0.399	0.704	0.519	0.722	0.717	0.609	0.625	0.619	0.695	0.689	**0.807**
21	0.421	0.508	0.450	0.559	0.575	0.618	**0.701**	0.484	0.566	0.483	0.624	0.652	0.696

**Table 3:** FAR and FDR of diesel engine obtained by different detection methods.

Sample Category	DPCA		DICA		DL-MB	LSW-IBI							
						Block1		Block2		Block3		Total	
	$T^{2}$	SPE	$I^{2}$	SPE	DR	$I^{2}$	SPE	$I^{2}$	SPE	$I^{2}$	SPE	$BIC\text{-}C^{2}$	BIC-SPE
Normal	0.061	0.061	0.068	0.031	0.065	0.077	0.069	0.073	0.059	0.067	0.062	0.120	0.086
Fault	0.706	0.824	0.758	0.860	0.862	0.564	0.773	0.805	0.869	0.698	0.869	0.817	0.919

## Data Availability

Data will be shared when there is a demand.
